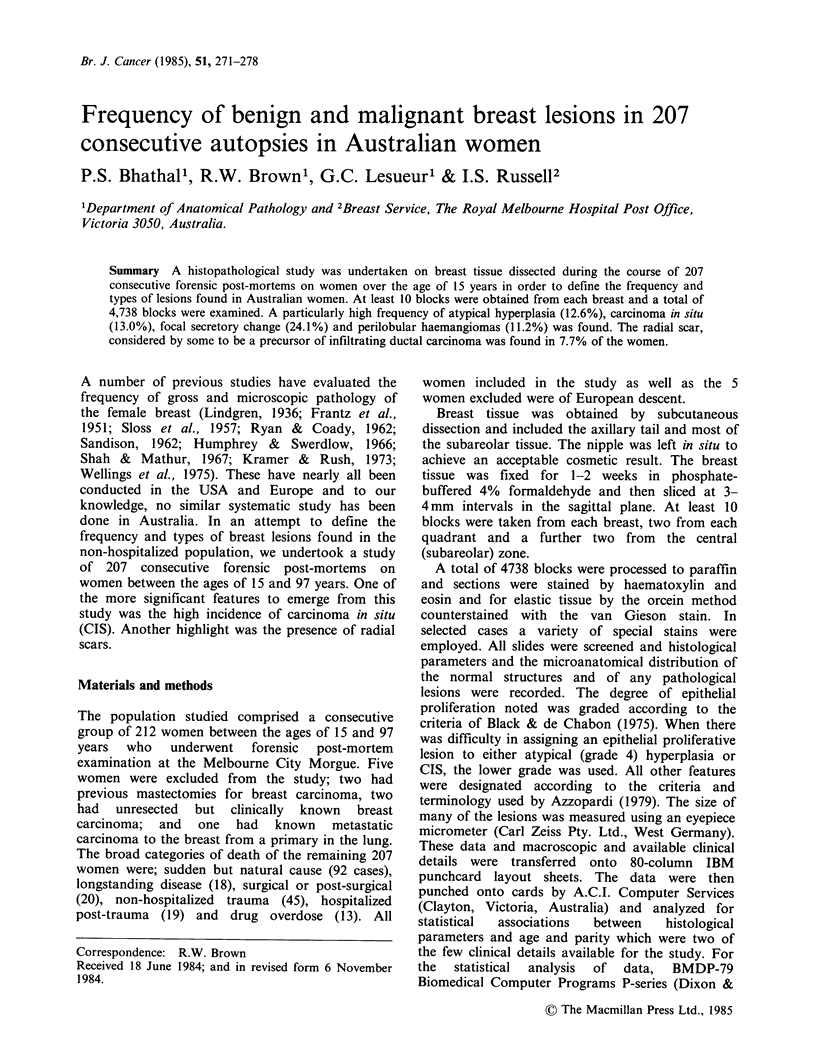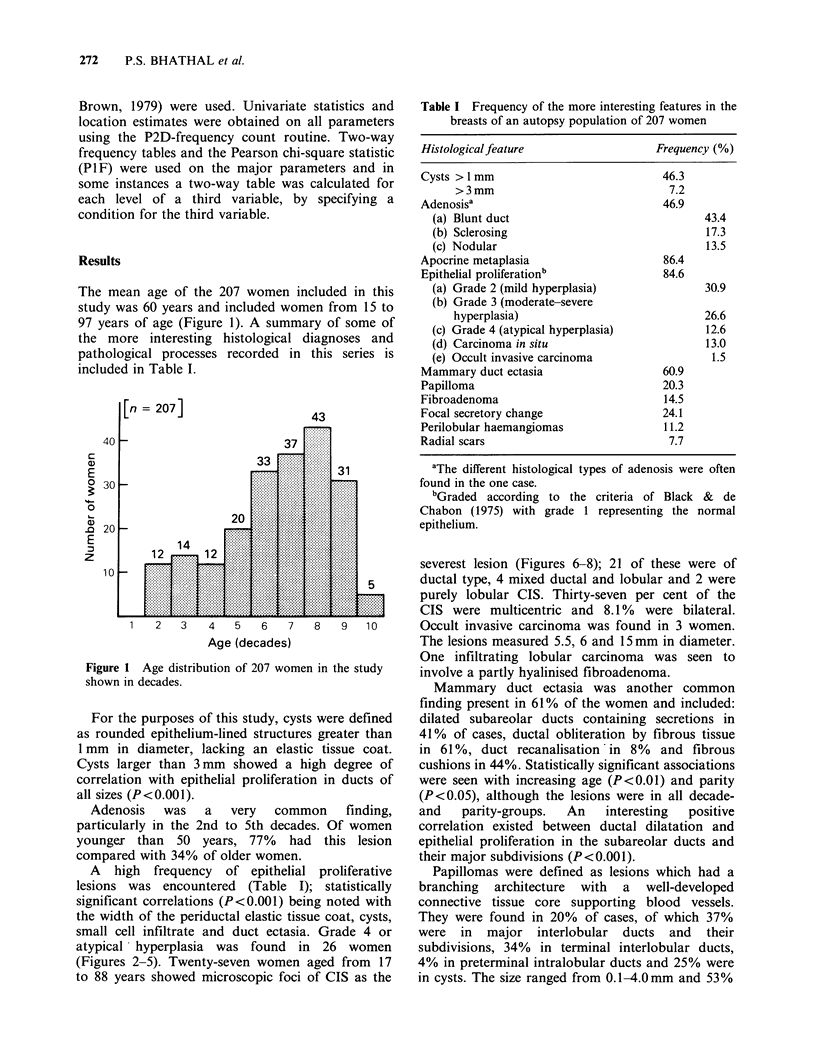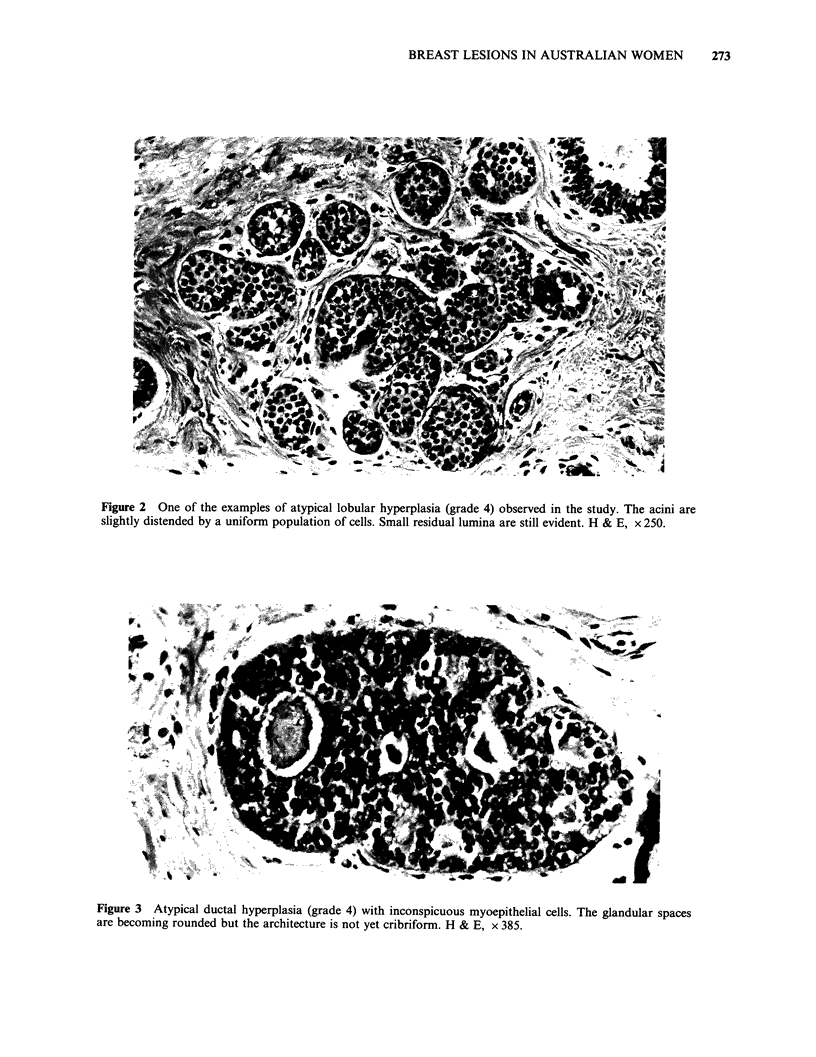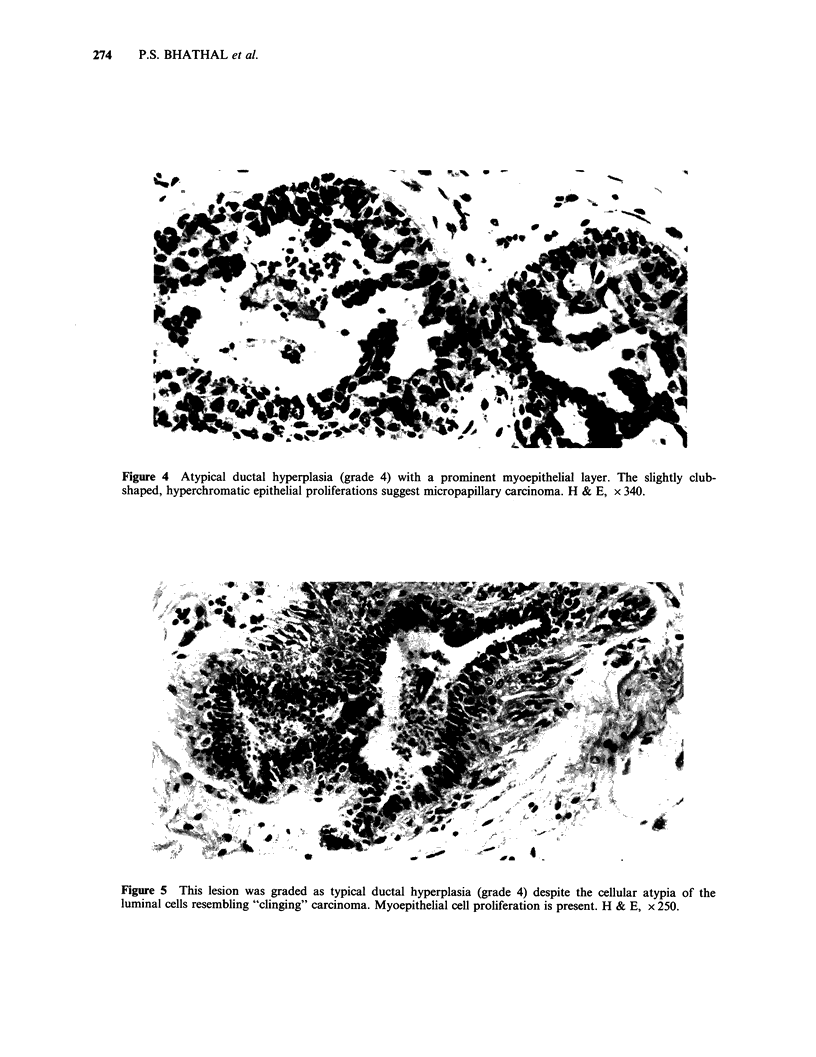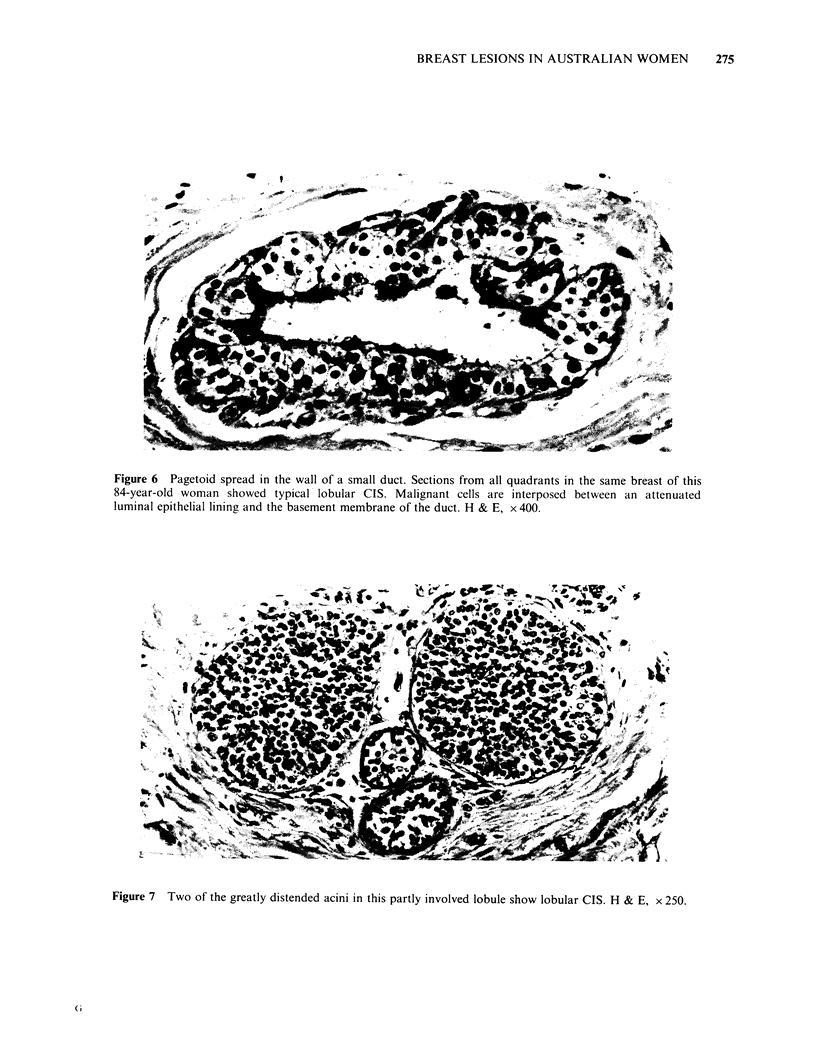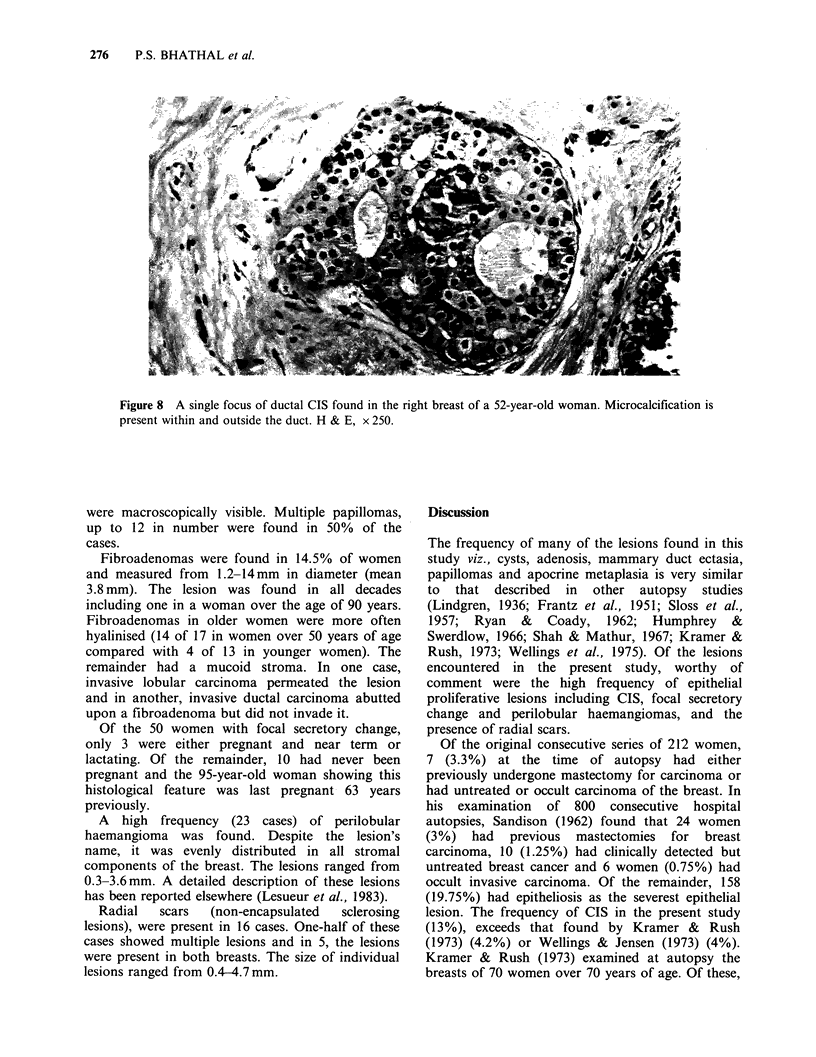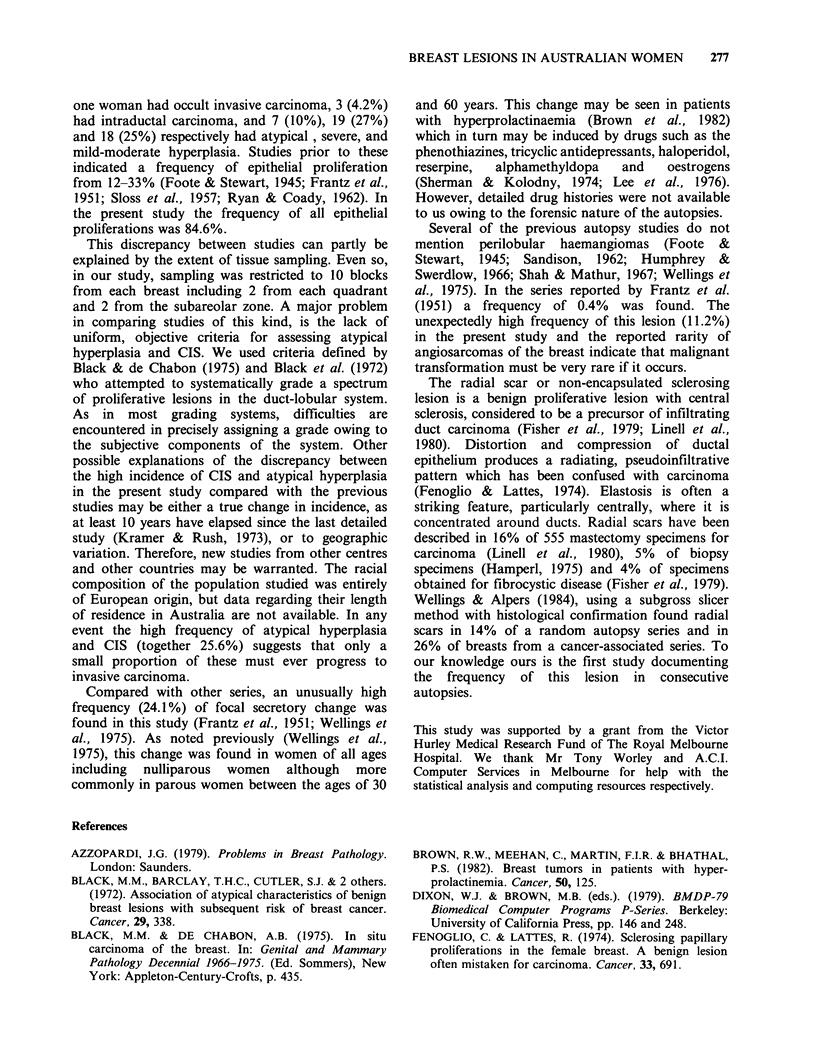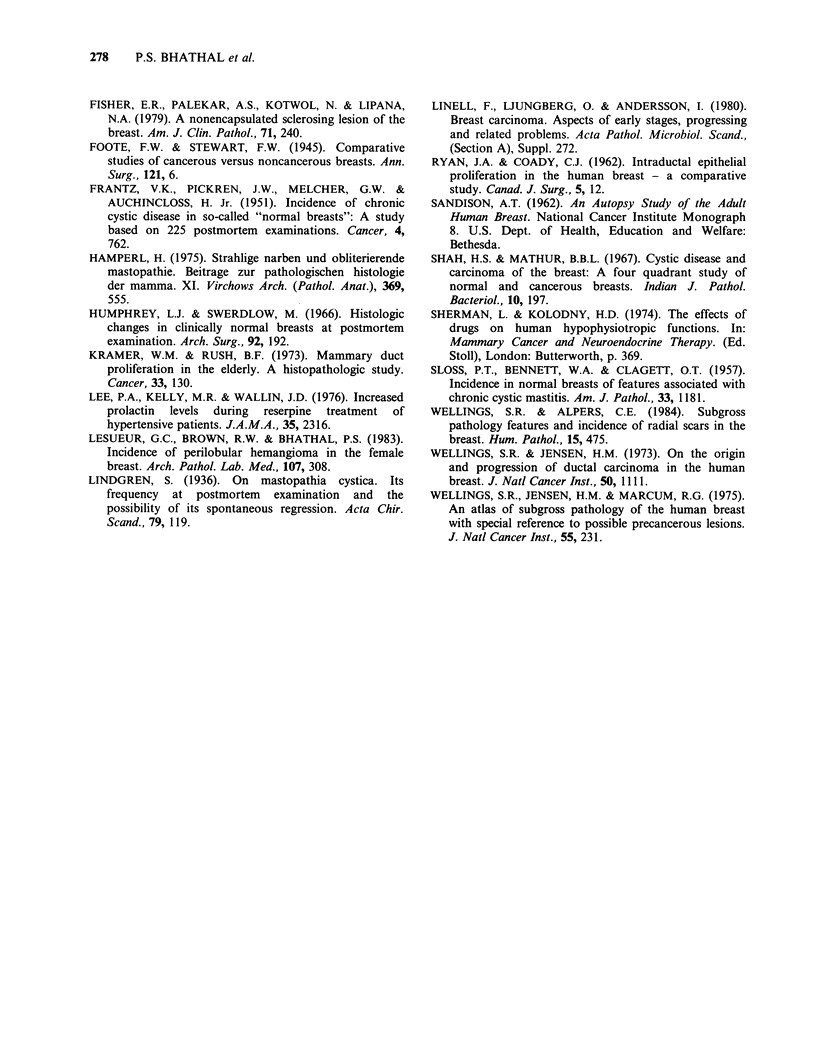# Frequency of benign and malignant breast lesions in 207 consecutive autopsies in Australian women.

**DOI:** 10.1038/bjc.1985.39

**Published:** 1985-02

**Authors:** P. S. Bhathal, R. W. Brown, G. C. Lesueur, I. S. Russell

## Abstract

**Images:**


*Br. J. Cancer* (1985), **51**, 271–278

### Frequency of benign and malignant breast lesions in 207 consecutive autopsies in Australian women

P.S. Bhathal[1], R.W. Brown[1], G.C. Lesueur[1] & I.S. Russell[2]

[1]*Department of Anatomical Pathology and* [2]*Breast Service, The Royal Melbourne Hospital Post Office, Victoria 3050, Australia.*

**Summary** A histopathological study was undertaken on breast tissue dissected during the course of 207 consecutive forensic post-mortems on women over the age of 15 years in order to define the frequency and types of lesions found in Australian women. At least 10 blocks were obtained from each breast and a total of 4,738 blocks were examined. A particularly high frequency of atypical hyperplasia (12.6%), carcinoma *in situ* (13.0%), focal secretory change (24.1%) and perilobular haemangiomas (11.2%) was found. The radial scar, considered by some to be a precursor of infiltrating ductal carcinoma was found in 7.7% of the women.

A number of previous studies have evaluated the frequency of gross and microscopic pathology of the female breast (Lindgren, 1936; Frantz *et al.*, 1951; Sloss *et al.*, 1957; Ryan & Coady, 1962; Sandison, 1962; Humphrey & Swerdlow, 1966; Shah & Mathur, 1967; Kramer & Rush, 1973; Wellings *et al.*, 1975). These have nearly all been conducted in the USA and Europe and to our knowledge, no similar systematic study has been done in Australia. In an attempt to define the frequency and types of breast lesions found in the non-hospitalized population, we undertook a study of 207 consecutive forensic post-mortems on women between the ages of 15 and 97 years. One of the more significant features to emerge from this study was the high incidence of carcinoma *in situ* (CIS). Another highlight was the presence of radial scars.

### Materials and methods

The population studied comprised a consecutive group of 212 women between the ages of 15 and 97 years who underwent forensic post-mortem examination at the Melbourne City Morgue. Five women were excluded from the study; two had previous mastectomies for breast carcinoma, two had unresected but clinically known breast carcinoma; and one had known metastatic carcinoma to the breast from a primary in the lung. The broad categories of death of the remaining 207 women were; sudden but natural cause (92 cases), longstanding disease (18), surgical or post-surgical (20), non-hospitalized trauma (45), hospitalized post-trauma (19) and drug overdose (13). All women included in the study as well as the 5 women excluded were of European descent.

Breast tissue was obtained by subcutaneous dissection and included the axillary tail and most of the subareolar tissue. The nipple was left *in situ* to achieve an acceptable cosmetic result. The breast tissue was fixed for 1–2 weeks in phosphate-buffered 4% formaldehyde and then sliced at 3–4 mm intervals in the sagittal plane. At least 10 blocks were taken from each breast, two from each quadrant and a further two from the central (subareolar) zone.

A total of 4738 blocks were processed to paraffin and sections were stained by haematoxylin and eosin and for elastic tissue by the orcein method counterstained with the van Gieson stain. In selected cases a variety of special stains were employed. All slides were screened and histological parameters and the microanatomical distribution of the normal structures and of any pathological lesions were recorded. The degree of epithelial proliferation noted was graded according to the criteria of Black & de Chabon (1975). When there was difficulty in assigning an epithelial proliferative lesion to either atypical (grade 4) hyperplasia or CIS, the lower grade was used. All other features were designated according to the criteria and terminology used by Azzopardi (1979). The size of many of the lesions was measured using an eyepiece micrometer (Carl Zeiss Pty. Ltd., West Germany). These data and macroscopic and available clinical details were transferred onto 80-column IBM punchcard layout sheets. The data were then punched onto cards by A.C.I. Computer Services (Clayton, Victoria, Australia) and analyzed for statistical associations between histological parameters and age and parity which were two of the few clinical details available for the study. For the statistical analysis of data, BMDP-79 Biomedical Computer Programs P-series (Dixon &

Correspondence: R.W. Brown
Received 18 June 1984; and in revised form 6 November 1984.

© The Macmillan Press Ltd., 1985

Brown, 1979) were used. Univariate statistics and location estimates were obtained on all parameters using the P2D-frequency count routine. Two-way frequency tables and the Pearson chi-square statistic (P1F) were used on the major parameters and in some instances a two-way table was calculated for each level of a third variable, by specifying a condition for the third variable.

### Results

The mean age of the 207 women included in this study was 60 years and included women from 15 to 97 years of age (Figure 1). A summary of some of the more interesting histological diagnoses and pathological processes recorded in this series is included in Table I.

**Figure 1** Age distribution of 207 women in the study shown in decades.

For the purposes of this study, cysts were defined as rounded epithelium-lined structures greater than 1 mm in diameter, lacking an elastic tissue coat. Cysts larger than 3 mm showed a high degree of correlation with epithelial proliferation in ducts of all sizes ($P<0.001$).

Adenosis was a very common finding, particularly in the 2nd to 5th decades. Of women younger than 50 years, 77% had this lesion compared with 34% of older women.

A high frequency of epithelial proliferative lesions was encountered (Table I); statistically significant correlations ($P<0.001$) being noted with the width of the periductal elastic tissue coat, cysts, small cell infiltrate and duct ectasia. Grade 4 or atypical hyperplasia was found in 26 women (Figures 2–5). Twenty-seven women aged from 17 to 88 years showed microscopic foci of CIS as the

**Table I** Frequency of the more interesting features in the breasts of an autopsy population of 207 women

| *Histological feature* | *Frequency* (%) | |
|---|---|---|
| Cysts >1 mm | 46.3 | |
| >3 mm | 7.2 | |
| Adenosis[a] | 46.9 | |
| (a) Blunt duct | | 43.4 |
| (b) Sclerosing | | 17.3 |
| (c) Nodular | | 13.5 |
| Apocrine metaplasia | 86.4 | |
| Epithelial proliferation[b] | 84.6 | |
| (a) Grade 2 (mild hyperplasia) | | 30.9 |
| (b) Grade 3 (moderate–severe hyperplasia) | | 26.6 |
| (c) Grade 4 (atypical hyperplasia) | | 12.6 |
| (d) Carcinoma *in situ* | | 13.0 |
| (e) Occult invasive carcinoma | | 1.5 |
| Mammary duct ectasia | 60.9 | |
| Papilloma | 20.3 | |
| Fibroadenoma | 14.5 | |
| Focal secretory change | 24.1 | |
| Perilobular haemangiomas | 11.2 | |
| Radial scars | 7.7 | |

[a]The different histological types of adenosis were often found in the one case.

[b]Graded according to the criteria of Black & de Chabon (1975) with grade 1 representing the normal epithelium.

severest lesion (Figures 6–8); 21 of these were of ductal type, 4 mixed ductal and lobular and 2 were purely lobular CIS. Thirty-seven per cent of the CIS were multicentric and 8.1% were bilateral. Occult invasive carcinoma was found in 3 women. The lesions measured 5.5, 6 and 15 mm in diameter. One infiltrating lobular carcinoma was seen to involve a partly hyalinised fibroadenoma.

Mammary duct ectasia was another common finding present in 61% of the women and included: dilated subareolar ducts containing secretions in 41% of cases, ductal obliteration by fibrous tissue in 61%, duct recanalisation in 8% and fibrous cushions in 44%. Statistically significant associations were seen with increasing age ($P<0.01$) and parity ($P<0.05$), although the lesions were in all decade- and parity-groups. An interesting positive correlation existed between ductal dilatation and epithelial proliferation in the subareolar ducts and their major subdivisions ($P<0.001$).

Papillomas were defined as lesions which had a branching architecture with a well-developed connective tissue core supporting blood vessels. They were found in 20% of cases, of which 37% were in major interlobular ducts and their subdivisions, 34% in terminal interlobular ducts, 4% in preterminal intralobular ducts and 25% were in cysts. The size ranged from 0.1–4.0 mm and 53%

**Figure 2** One of the examples of atypical lobular hyperplasia (grade 4) observed in the study. The acini are slightly distended by a uniform population of cells. Small residual lumina are still evident. H & E, ×250.

**Figure 3** Atypical ductal hyperplasia (grade 4) with inconspicuous myoepithelial cells. The glandular spaces are becoming rounded but the architecture is not yet cribriform. H & E, ×385.

**Figure 4** Atypical ductal hyperplasia (grade 4) with a prominent myoepithelial layer. The slightly club-shaped, hyperchromatic epithelial proliferations suggest micropapillary carcinoma. H & E, ×340.

**Figure 5** This lesion was graded as typical ductal hyperplasia (grade 4) despite the cellular atypia of the luminal cells resembling "clinging" carcinoma. Myoepithelial cell proliferation is present. H & E, ×250.

**Figure 6** Pagetoid spread in the wall of a small duct. Sections from all quadrants in the same breast of this 84-year-old woman showed typical lobular CIS. Malignant cells are interposed between an attenuated luminal epithelial lining and the basement membrane of the duct. H & E, ×400.

**Figure 7** Two of the greatly distended acini in this partly involved lobule show lobular CIS. H & E, ×250.

**Figure 8** A single focus of ductal CIS found in the right breast of a 52-year-old woman. Microcalcification is present within and outside the duct. H & E, ×250.

were macroscopically visible. Multiple papillomas, up to 12 in number were found in 50% of the cases.

Fibroadenomas were found in 14.5% of women and measured from 1.2–14 mm in diameter (mean 3.8 mm). The lesion was found in all decades including one in a woman over the age of 90 years. Fibroadenomas in older women were more often hyalinised (14 of 17 in women over 50 years of age compared with 4 of 13 in younger women). The remainder had a mucoid stroma. In one case, invasive lobular carcinoma permeated the lesion and in another, invasive ductal carcinoma abutted upon a fibroadenoma but did not invade it.

Of the 50 women with focal secretory change, only 3 were either pregnant and near term or lactating. Of the remainder, 10 had never been pregnant and the 95-year-old woman showing this histological feature was last pregnant 63 years previously.

A high frequency (23 cases) of perilobular haemangioma was found. Despite the lesion's name, it was evenly distributed in all stromal components of the breast. The lesions ranged from 0.3–3.6 mm. A detailed description of these lesions has been reported elsewhere (Lesueur *et al.*, 1983).

Radial scars (non-encapsulated sclerosing lesions), were present in 16 cases. One-half of these cases showed multiple lesions and in 5, the lesions were present in both breasts. The size of individual lesions ranged from 0.4–4.7 mm.

### Discussion

The frequency of many of the lesions found in this study *viz.*, cysts, adenosis, mammary duct ectasia, papillomas and apocrine metaplasia is very similar to that described in other autopsy studies (Lindgren, 1936; Frantz *et al.*, 1951; Sloss *et al.*, 1957; Ryan & Coady, 1962; Humphrey & Swerdlow, 1966; Shah & Mathur, 1967; Kramer & Rush, 1973; Wellings *et al.*, 1975). Of the lesions encountered in the present study, worthy of comment were the high frequency of epithelial proliferative lesions including CIS, focal secretory change and perilobular haemangiomas, and the presence of radial scars.

Of the original consecutive series of 212 women, 7 (3.3%) at the time of autopsy had either previously undergone mastectomy for carcinoma or had untreated or occult carcinoma of the breast. In his examination of 800 consecutive hospital autopsies, Sandison (1962) found that 24 women (3%) had previous mastectomies for breast carcinoma, 10 (1.25%) had clinically detected but untreated breast cancer and 6 women (0.75%) had occult invasive carcinoma. Of the remainder, 158 (19.75%) had epitheliosis as the severest epithelial lesion. The frequency of CIS in the present study (13%), exceeds that found by Kramer & Rush (1973) (4.2%) or Wellings & Jensen (1973) (4%). Kramer & Rush (1973) examined at autopsy the breasts of 70 women over 70 years of age. Of these, one woman had occult invasive carcinoma, 3 (4.2%) had intraductal carcinoma, and 7 (10%), 19 (27%) and 18 (25%) respectively had atypical , severe, and mild-moderate hyperplasia. Studies prior to these indicated a frequency of epithelial proliferation from 12–33% (Foote & Stewart, 1945; Frantz *et al.*, 1951; Sloss *et al.*, 1957; Ryan & Coady, 1962). In the present study the frequency of all epithelial proliferations was 84.6%.

This discrepancy between studies can partly be explained by the extent of tissue sampling. Even so, in our study, sampling was restricted to 10 blocks from each breast including 2 from each quadrant and 2 from the subareolar zone. A major problem in comparing studies of this kind, is the lack of uniform, objective criteria for assessing atypical hyperplasia and CIS. We used criteria defined by Black & de Chabon (1975) and Black *et al.* (1972) who attempted to systematically grade a spectrum of proliferative lesions in the duct-lobular system. As in most grading systems, difficulties are encountered in precisely assigning a grade owing to the subjective components of the system. Other possible explanations of the discrepancy between the high incidence of CIS and atypical hyperplasia in the present study compared with the previous studies may be either a true change in incidence, as at least 10 years have elapsed since the last detailed study (Kramer & Rush, 1973), or to geographic variation. Therefore, new studies from other centres and other countries may be warranted. The racial composition of the population studied was entirely of European origin, but data regarding their length of residence in Australia are not available. In any event the high frequency of atypical hyperplasia and CIS (together 25.6%) suggests that only a small proportion of these must ever progress to invasive carcinoma.

Compared with other series, an unusually high frequency (24.1%) of focal secretory change was found in this study (Frantz *et al.*, 1951; Wellings *et al.*, 1975). As noted previously (Wellings *et al.*, 1975), this change was found in women of all ages including nulliparous women although more commonly in parous women between the ages of 30 and 60 years. This change may be seen in patients with hyperprolactinaemia (Brown *et al.*, 1982) which in turn may be induced by drugs such as the phenothiazines, tricyclic antidepressants, haloperidol, reserpine, alphamethyldopa and oestrogens (Sherman & Kolodny, 1974; Lee *et al.*, 1976). However, detailed drug histories were not available to us owing to the forensic nature of the autopsies.

Several of the previous autopsy studies do not mention perilobular haemangiomas (Foote & Stewart, 1945; Sandison, 1962; Humphrey & Swerdlow, 1966; Shah & Mathur, 1967; Wellings *et al.*, 1975). In the series reported by Frantz *et al.* (1951) a frequency of 0.4% was found. The unexpectedly high frequency of this lesion (11.2%) in the present study and the reported rarity of angiosarcomas of the breast indicate that malignant transformation must be very rare if it occurs.

The radial scar or non-encapsulated sclerosing lesion is a benign proliferative lesion with central sclerosis, considered to be a precursor of infiltrating duct carcinoma (Fisher *et al.*, 1979; Linell *et al.*, 1980). Distortion and compression of ductal epithelium produces a radiating, pseudoinfiltrative pattern which has been confused with carcinoma (Fenoglio & Lattes, 1974). Elastosis is often a striking feature, particularly centrally, where it is concentrated around ducts. Radial scars have been described in 16% of 555 mastectomy specimens for carcinoma (Linell *et al.*, 1980), 5% of biopsy specimens (Hamperl, 1975) and 4% of specimens obtained for fibrocystic disease (Fisher *et al.*, 1979). Wellings & Alpers (1984), using a subgross slicer method with histological confirmation found radial scars in 14% of a random autopsy series and in 26% of breasts from a cancer-associated series. To our knowledge ours is the first study documenting the frequency of this lesion in consecutive autopsies.

This study was supported by a grant from the Victor Hurley Medical Research Fund of The Royal Melbourne Hospital. We thank Mr Tony Worley and A.C.I. Computer Services in Melbourne for help with the statistical analysis and computing resources respectively.

### References

AZZOPARDI, J.G. (1979). *Problems in Breast Pathology*. London: Saunders.

BLACK, M.M., BARCLAY, T.H.C., CUTLER, S.J. & 2 others. (1972). Association of atypical characteristics of benign breast lesions with subsequent risk of breast cancer. *Cancer*, **29**, 338.

BLACK, M.M. & DE CHABON, A.B. (1975). In situ carcinoma of the breast. In: *Genital and Mammary Pathology Decennial 1966–1975*. (Ed. Sommers), New York: Appleton-Century-Crofts, p. 435.

BROWN, R.W., MEEHAN, C., MARTIN, F.I.R. & BHATHAL, P.S. (1982). Breast tumors in patients with hyper-prolactinemia. *Cancer*, **50**, 125.

DIXON, W.J. & BROWN, M.B. (eds.). (1979). *BMDP-79 Biomedical Computer Programs P-Series*. Berkeley: University of California Press, pp. 146 and 248.

FENOGLIO, C. & LATTES, R. (1974). Sclerosing papillary proliferations in the female breast. A benign lesion often mistaken for carcinoma. *Cancer*, **33**, 691.

FISHER, E.R., PALEKAR, A.S., KOTWOL, N. & LIPANA, N.A. (1979). A nonencapsulated sclerosing lesion of the breast. *Am. J. Clin. Pathol.*, **71**, 240.

FOOTE, F.W. & STEWART, F.W. (1945). Comparative studies of cancerous versus noncancerous breasts. *Ann. Surg.*, **121**, 6.

FRANTZ, V.K., PICKREN, J.W., MELCHER, G.W. & AUCHINCLOSS, H. Jr. (1951). Incidence of chronic cystic disease in so-called "normal breasts": A study based on 225 postmortem examinations. *Cancer*, **4**, 762.

HAMPERL, H. (1975). Strahlige narben und obliterierende mastopathie. Beitrage zur pathologischen histologie der mamma. XI. *Virchows Arch.* (*Pathol. Anat.*), **369**, 555.

HUMPHREY, L.J. & SWERDLOW, M. (1966). Histologic changes in clinically normal breasts at postmortem examination. *Arch. Surg.*, **92**, 192.

KRAMER, W.M. & RUSH, B.F. (1973). Mammary duct proliferation in the elderly. A histopathologic study. *Cancer*, **33**, 130.

LEE, P.A., KELLY, M.R. & WALLIN, J.D. (1976). Increased prolactin levels during reserpine treatment of hypertensive patients. *J.A.M.A.*, **35**, 2316.

LESUEUR, G.C., BROWN, R.W. & BHATHAL, P.S. (1983). Incidence of perilobular hemangioma in the female breast. *Arch. Pathol. Lab. Med.*, **107**, 308.

LINDGREN, S. (1936). On mastopathia cystica. Its frequency at postmortem examination and the possibility of its spontaneous regression. *Acta Chir. Scand.*, **79**, 119.

LINELL, F., LJUNGBERG, O. & ANDERSSON, I. (1980). Breast carcinoma. Aspects of early stages, progressing and related problems. *Acta Pathol. Microbiol. Scand.*, (Section A), Suppl. 272.

RYAN, J.A. & COADY, C.J. (1962). Intraductal epithelial proliferation in the human breast – a comparative study. *Canad. J. Surg.*, **5**, 12.

SANDISON, A.T. (1962). *An Autopsy Study of the Adult Human Breast.* National Cancer Institute Monograph 8. U.S. Dept. of Health, Education and Welfare: Bethesda.

SHAH, H.S. & MATHUR, B.B.L. (1967). Cystic disease and carcinoma of the breast: A four quadrant study of normal and cancerous breasts. *Indian J. Pathol. Bacteriol.*, **10**, 197.

SHERMAN, L. & KOLODNY, H.D. (1974). The effects of drugs on human hypophysiotropic functions. In: *Mammary Cancer and Neuroendocrine Therapy*. (Ed. Stoll), London: Butterworth, p. 369.

SLOSS, P.T., BENNETT, W.A. & CLAGETT, O.T. (1957). Incidence in normal breasts of features associated with chronic cystic mastitis. *Am. J. Pathol.*, **33**, 1181.

WELLINGS, S.R. & ALPERS, C.E. (1984). Subgross pathology features and incidence of radial scars in the breast. *Hum. Pathol.*, **15**, 475.

WELLINGS, S.R. & JENSEN, H.M. (1973). On the origin and progression of ductal carcinoma in the human breast. *J. Natl Cancer Inst.*, **50**, 1111.

WELLINGS, S.R., JENSEN, H.M. & MARCUM, R.G. (1975). An atlas of subgross pathology of the human breast with special reference to possible precancerous lesions. *J. Natl Cancer Inst.*, **55**, 231.